# Decadal trends in illicit drug-related poisonings in Morocco: Epidemiological analysis and public health implications

**DOI:** 10.1192/j.eurpsy.2025.1482

**Published:** 2025-08-26

**Authors:** O. Erefai, S. Hmimou, S. Elkafssaoui, H. Harifi, A. Soulaymani, A. Mokhtari, H. Hami

**Affiliations:** 1 Higher Institute of Nursing Professions and Health Techniques, Rabat; 2 Laboratory of Biology and Health, Faculty of Science, Ibn Tofail University, Kenitra; 3 Royal School of Military Health Service, Rabat, Morocco

## Abstract

**Introduction:**

The impact of **illicit** drug-related poisonings on public health is a pressing global concern, necessitating focused scrutiny to mitigate adverse effects on communities. In Morocco, the prevalence of **illicit** substance abuse and its resulting emergencies pose a complex challenge to public health systems. **Particularly concerning are the poisonings induced by these substances, which not only require immediate responses but also demand a sustained, strategic approach to ensure long-term community** health **and safety**.

**Objectives:**

This study aims to delineate trends in poisonings caused by illicit drugs, as reported to the Moroccan Poison Control Center over a decade, focusing on the annual number of cases and their proportion to overall poisoning cases.

**Methods:**

Data spanning from 2013 to 2022 from the Moroccan Poison Control Center were analyzed to assess the annual number of poisoning cases and proportions attributed to incidents involving illicit drug use. The analysis focused on identifying long-term trends, highlighting the evolving role of **illicit** drugs in poisoning statistics.

**Results:**

Between 2013 and 2022, a total of 109,976 poisoning cases were recorded, of which 2,284 (2.08%) were related to the use of illicit drugs. Initially, drug-related poisonings increased, peaking in 2017 at 339 cases (2% of 16,906 total cases). Notable observations included 198 drug-related cases in 2013 (1.65% of 11,968 cases) and 334 in 2016 (2.11% of 15,838 cases). However, a marked decline was observed post-2017, culminating in 2022 with 84 drug-related cases (1.99% of 4,225 total cases). The prevalence of drug-related poisonings fluctuated annually, ranging from 1.56% to 3.65%, with a significant decrease in both total and drug-related poisonings after 2017.

**Conclusions:**

Although there was an initial increase in **illicit** drug-related poisonings in Morocco, a notable decline was observed. Despite this downturn, **illicit** drug-related poisonings continue to pose a critical public health issue. Ongoing monitoring and proactive prevention strategies are crucial to sustain this downward trend and effectively manage the challenges of illicit substance abuse.

**Disclosure of Interest:**

None Declared

